# Detection of human papillomavirus genotypes, herpes simplex, varicella zoster and cytomegalovirus in breast cancer patients

**DOI:** 10.1186/s12985-021-01498-z

**Published:** 2021-01-22

**Authors:** Morvarid Golrokh Mofrad, Zohreh Azita Sadigh, Sanaz Ainechi, Ebrahim Faghihloo

**Affiliations:** 1 Razi Vaccine and Serum Research Institute, Agricultural Research, Education and Extension Organization (AREEO), Karaj, Iran; 2 Human Viral Vaccine Department, Razi Vaccine and Serum Research Institute, Agricultural Research, Education and Extension Organization (AREEO), Karaj, Iran; 3 Department of Pathology, Keck School of Medicine, University of Southern California, Los Angeles, CA USA; 4 Department of Microbiology, School of Medicine, Shahid Beheshti University of Medical Sciences, Tehran, Iran

**Keywords:** Human papillomavirus, HPV, Breast cancer, Genotype, Herpes virus, PCR

## Abstract

**Background:**

The role of viruses as a cause of breast cancer (BC) has been significantly investigated in recent years. Human papillomavirus (HPV) has been detected in invasive breast carcinomas, while most studies have only focused on the detection of viral DNA, we aimed to examine the prevalence and genotypes of HPV among Iranian BC patients.

We also examined the presence of herpes simplex-1 (HSV-1), herpes simplex-2 (HSV-2), varicella zoster virus (VZV), and cytomegalovirus (CMV) in these samples.

**Methods:**

We collected and analyzed 70 Formalin-Fixed Paraffin-Embedded (FFPE) blocks including 59 BC samples, and 11 benign breast lesions as control from Iranian patients using nested PCR. Real-time PCR utilized as a confirming test to nested PCR findings. Genotyping of HPV positive samples was performed, the samples were also subjected to a multiplex PCR to detect HSV-1, HSV-2, VZV, and CMV in BC.

**Results:**

Papillomavirus DNA was present in 7 of 59 BC samples (11.8%); while none was detected in control samples. The most prevalent type was HPV18, followed by HPV 6. All HPV positive patients had high tumor grades (II/ III) with a histologic diagnosis of ductal carcinoma. The patient age range was 33 to 73 years with a median of 51 years. Most of HPV positive patients had low levels of education. HPV16 was not detected. Also, 5 of 59 BC specimens (8.47%), were positive for HSV-1. But none of the samples were positive for HSV-2, VZV, and CMV.

**Conclusions:**

Our results suggest a carcinogenesis role for High-risk HPV (HPV18) in breast tumors. Our findings of HSV-1 and low-risk HPV (HPV6) in BCs may propose a cancer-causing role for them. Further large-scale studies are warranted to assess the significance of our findings.

## Background

Breast Cancer (BC) is the most prevalent cancer worldwide among women. BC accounts for approximately 30% of female cancers and is the most frequent cause of death in women [1, 2]. Over the last decade incidence rates of BC have raised by around 20% worldwide. Even though the incidence rate of BC is higher in developed countries, has higher mortality in developing countries. More than 55% of breast cancer-related deaths occur in them [3, 4]. Lack of early detection programs, inadequate diagnostic and treatment facilities are the main reasons for the low survival rate in most developing countries [5, 6]. The fifth most common cancer-related death in Iran is BC [7]. Different factors, such as family history, genetics, decreased childbearing and breastfeeding, harmful dietary, previous benign breast disease, radiation, oral contraceptives, smoking, and alcohol intake are considered as risk factors in the onset of cancer [8–10]. Meanwhile, a pathogenesis role for some infectious agents including viruses has been questioned [11–14].

Some studies have shown the role of human papillomavirus (HPV) in different cancers [15–17]. Over the past two decades, various studies have suggested a role for HPV in breast cancer [18–22].

Thus far over 200 HPV types have been identified and most of them fall within three genera: alpha (α), beta (β), and gamma (γ). While alpha type (α) is mainly found on mucosal surfaces, beta (β) and gamma (γ) types are usually detected on the skin surface and in hair follicles [23, 24]. HPVs are classified into low and high-risk types based on their oncogenic potential [25]. Low-risk HPVs as the main pathogen in benign genital warts are not usually associated with any other neoplasms. Low risk HPVs include: 6, 11, 34, 40, 42, 43, 44, 53, 54, 66, 70, and 74. High risk HPV types named for their carcinogenicity, and comprised of 16, 18, 31, 33, 35, 39, 45, 51, 52, 56, 58, 59, 68, and 69 [26, 27]. For the first time in 1992, Di Lonardo A and colleagues suggested that HPV could play a crucial role in the genesis of breast carcinoma by detecting HPV16 DNA sequence in 29.4% of breast and lymph node samples [28]. Following the first publication, several more studies have documented the presence of HPV in malignant breast tumors [29–31]. On the contrary negative relationship between HPV and breast cancer has been declared by a few studies [32–34]. Therefore, the association between HPV and breast carcinogenesis is not completely clear. Due to the high rate of breast cancer in Iranian women and the probable role of high-risk HPV in its carcinogenesis, we determined to investigate the presence of two high-risk genotypes; HPV16 and HPV18 in our study group.

Besides, we decided to investigate the presence of herpesviruses (HSV1, HSV2, VZV, and CMV) in our collected BC cases, as some studies have reported a relation between a group of herpesviruses and breast cancer [35–39].

## Methods

### Sample collection

This cross-sectional, case–control study was conducted in Modarres hospital, Tehran, Iran, on cases archived between 2008 and 2019 years. A total of 70 blocks of Formalin-Fixed Paraffin-Embedded (FFPE) including 59 samples diagnosed as breast carcinomas, and 11 benign breast lesions as control were collected from the pathology department archives of Modarres hospital. Also, several parameters; such as type of BC, grading of breast cancer, age, and level of education were taken in written format as exclusion criteria and summarized in Table 1. All study procedures are performed in laboratories of Shahid Beheshti Medical School of sciences.

**Table 1:** Clinical and pathological features of all cases

	Tumor cases	Control cases
Numbers	59	11
Age ranges	29 to 81 years	33 to 69 years
Histological types	5% Lobular	
	95% Ductal	Benign breast lesions
Grade	6/8% Low grade	
	93/2% High grade	–
Levels of education	56% Low level of education	54% Low level of education
	24% High level of education	46% high level of education
	20% No education	

*L* low grade (I), *H* high grade (II/ III)

### DNA extraction

The genomic DNA was extracted from FFPE breast tissues. Then we performed a standard polymerase chain reaction (PCR) to detect HPV DNA. To extract DNA, FFPE tissues were cut in 10 μm thickness by a microtome. Deparaffinization was performed by adding 1 ml xylene and spinning. Afterwards, we centrifuged the samples for 5 min, and supernatants were removed. This step was repeated once, then 1 ml of 96% ethanol was added. Microtubes were put in a 50ºC heating block until ethanol was entirely dried up. Then digestion was performed by adding digestion buffer and proteinase K solution. Later, the microtubes were incubated overnight, and the next day they were placed in a 95 ºC heater. Respectively phenol, phenol–chloroform, and chloroform solutions were added to samples, each step followed by centrifugation, and subsequent removal of the supernatant. Ultimately, ethanol was added, then they were kept overnight in an incubator.

Samples were centrifuged at 12,000*g*$\times$ for 30 min on the last day of the extraction. After removing the supernatant, the uncapped microtubes were left in a 37 °C heater to let the residual ethanol to evaporate. Finally, distilled water was added and the DNA extracts were quantified with a NanoDrop spectrophotometer.

The quality of DNA extracted from paraffin-embedded tissue samples was assessed by a forward (ATGTTCGTCATGGGTGTGAA) and reverse (GGTGCTAAGCAGTTGGTGGT) primer pair targeting a sequence within the GAPDH gene. PCR amplification protocol consisted of 30 cycles of denaturation at 95 °C for 30 s; hybridization at 55 °C for 30 s, and elongation at 72 °C for 30 s. A final elongation step was performed at 72 °C for 10 min.

### PCR for HPV and herpes viruses

All samples were screened for HPV L1 conserved region. The nested PCR assay was performed using two sets of primers (MY09/11 and GP5+ /6+) for two consecutive amplification reactions. The first reaction was performed in 25 μl using 12.5 μl of master mix (which includes:1X PCR buffer, 2 mM MgCl_2_, 50 μM of each deoxynucleotide triphosphate (dNTP) and 2 U of Taq DNA polymerase (Takapouzist, Iran), 100–200 ng of template DNA, 10 pmol of each consensus outer degenerate primer MY09 (5′-CGTCC(A/C)A(A/G)(A/G)GGA(A/T)ACTGATC -3′) /MY11(5′-GC(A/C)CAGGG(A/T)CTATAA(C/T)AATGG -3′) and distilled water. Thermal cycling (Bio Intellectica) performed with the following program: 5 min at 94 °C, 40 cycles of 1 min at 94 °C, 1 min at 55 °C, and 1 min at 72 °C, with a final extension step at 72 °C for 7 min.

The second reaction was also performed in 50 μl including 25 μl of master mix (which contains: 1X PCR buffer, 3 mM MgCl_2_, 50 μM each dNTP, 2U Taq DNA polymerase), 100–200 ng of amplified DNA, and 10 pmol each inner consensus primer GP5** + **(5′- TTTGTTACTGTGGTAGATACTAC-3′) and GP6 + (5′-AAAAATAAACTGTAAATCATATTC-3′), and distilled water. Thermal cycling used the following program: 4 min at 94 °C, 40 cycles of 1 min at 94 °C, 2 min at 40 °C, and 2 min at 72 °C, with a final extension step at 72 °C for 4 min.

Negative controls containing water instead of DNA were used. We used the HPV18 HeLa cell line as a positive control.

Real-time PCR amplification was performed in a 20 μl volume containing 4 μl 5 × HOT FIREPol® Probe qPCR Mix Plus (no ROX) (Solis BioDyne, Estonia), 0, 5 μl of each forward and reverse primers (Pishgam company, Tehran, Iran), 0.5 μl of probes (Sinaclon Co., Tehran, Iran) which listed in Table 2, and 2 μl of each sample or control, and rest of the total volume was obtained by adding distilled water. Amplification and detection were performed by a real-time PCR machine (Rotor-Gene-Q 6000 thermocycler (Corbett, Australia)). Thermal cycles used for quantification of HPV18 E6 gene and HPV type 16 E7 gene were 95 °C for 12 min, 95 °C for 15 s, and 60 °C for 60 s for 40 cycles.

**Table 2:** List of primers used for GAPDH PCR, nested PCR, real-time PCR, and multiplex PCR

Assay	Name	Sequence (5′ → 3′)
GAPDH	GAPDH-F	ATGTTCGTCATGGGTGTGAA
(110 bp)	GAPDH-R	GGTGCTAAGCAGTTGGTGGT
MY09/11	MY09	CGTCC (A/C) A (A/G) (A/G) GGA (A/T) ACTGATC
(450 bp)	MY11	GC (A/C) CAGGG (A/T) CTATAA(C/T) AATGG
GP5 + /GP6 +	GP5 +	TTTGTTACTGTGGTAGATACTAC
(150 bp)	GP6 +	AAAAATAAACTGTAAATCATATTC
HPV 18 E6	HPV 18 E6-F	CTG GGC ACT ATA GAG GCC AGT
(78 bp)	HPV 18 E6-R	GTG TTT CTC TGC GTC GTT GG
	HPV 18 E6-P	[FAM] -TGCAACCGAGCACGACAGGAACGA—[TAMRA]
HPV 16 E7	HPV 16 E7-F	GAG GAG GAG GAT GAA ATA GAT GGT
(98 bp)	HPV 16 E7-R	AGC GTA GAG TCA CAC TTG CAA CA
	HPV 16 E7-P	[FAM] -CTCTGTCCGGTTCTGCTTGTCCAGCT- [TAMRA]
HSV-1	HSV-1-F	GACTCTCCCACCGCCATCAG
(269 bp)	HSV-1-R	TGTCTTCGGGCGACTGGT
HSV-2	HSV-2-F	TATGCCTATCCCCGGTTGGA
(715 bp)	HSV-2-R	CGTGCCATCCGAATAAACGTG
VZV	VZV-F	TTGTGTCGGTCTCTCCAAGC
(934 bp)	VZV-R	TACGTCTTCAACCTCACGCC
CMV	CMV-F	TGGCTTTTCTTGAACGTGCG
(716 bp)	CMV-R	CCTTGACGCTGGTTTGGTTG

*F* forward primer, *R* reverse primer, *P* probe, *FAM* 6-carboxy fluorescein, *TAMRA* 6-carboxy tetramethyl rhodamine, *bp* base pair

Duplicate reactions for each gene were performed. Probes were labeled with 6-FAM at the 5′ end and TAMRA at the 3′ end.

Each PCR was performed with negative (DNA free water) and positive controls (genomic DNA of SiHa cells for HPV-16, and genomic DNA of HeLa cells for HPV-18).

The samples were also subjected to a multiplex PCR to detect HSV-1, HSV-2, VZV, and CMV. The PCR reactions were performed in a total volume of 25 μL containing 12.5 μl PCR Master Mix (10X PCR Taq polymerase buffer, 10 mM of dNTPs and 1.5 U/rxn of Taq DNA polymerase [Takapouzist, Iran]), 10 pmol of each primer (HSV-1, HSV-2, CMV, and VZV) (Table 2), and 50 ng of genomic DNA.

mPCR (multiplex PCR) conditions were as follows: Initial denaturation (95 °C for 5 min), 35 cycles of denaturation (95 °C for 30 s) annealing (60 °C for 30 s) and extension (72 °C for 30 s); Final extension was given at 72 °C for 10 min. Samples were preserved at 4 °C and then the PCR product was detected by gel electrophoresis.

### Sequencing and phylogenetic analysis

The positive PCR samples were sequenced for HPV genotypes. The DNA sequence was determined with the Big-Dye terminator cycle sequencing kit and an ABI 377A sequencer (Applied Biosystems Inc.).

The HPV sequences were edited with the BioEdit program version 7.2.3, and then phylogenetic and molecular analyses were organized using MEGA software program version 6.0.6 [27]. The neighbor joining and the Kimura 2-parameters methods were used for phylogenetic reconstructions that were implemented in the MEGA 6.0.6 program. Statistical significance for the phylogenetic tree was assessed by the bootstrap method (1000 replicates).

Data were analyzed by SPSS statistical software program version 16.0. The correlations were subjected to *χ*^2^ (Pearson chi-square) and Fisher’s exact test. Odds ratios and logistic regression were also calculated. Statistical significance was set as a *P*-value less than 0.05.

### Deposit the nucleotide sequence

The nucleotide sequences of HPV isolates that were found out in this study have been settled in the GenBank database [Accession numbers QED55703–QED55709]. The GenBank accession numbers for HPV6 types are QED55703 and QED55704, and for HPV18 types are QED55705, QED55706, QED55707, QED55708, and QED55709.

## Results

### Study population

HPV gene sequences were identified. The patient age range was between 29 to 81 years (mean of 50 years). Only 4 patients had low-grade malignancy (6.7%), and the remaining were diagnosed with high grade (93.3%). Regarding histologic types, 5% of tumors were lobular types, and the remaining (95%) were ductal carcinomas (Table 3). We reviewed the level of education of patients and we found 24% with high education level (university degree), 56% with low education level (high school diploma or less), and 20% with no education.

**Table 3:** Clinical and pathological features of positive cases

	Tumor cases	Control cases	Tumor grade	Histological type
			High grade (II/III)	Lobular/ ductal
Total cases	59	11	93/2% High grade	5% Lobular, and 95% Ductal
HPV-positive	7	0	100% High grade	100% Ductal
HPV-negative	52	11		
HSV-1-positive	5	0	80% High grade	100% Ductal
HSV-1-negative	54	11		
HSV-2-positive	0	0	–	–
HSV-2-negative	59	11	–	–
VZV-positive	0	0	–	–
VZV-negative	59	11	–	–
CMV-positive	0	0	–	–
CMV-negative	59	11	–	–

*L* low grade (I), *H* high grade (II/ III)

### Assessing DNA quality

**Fig. 1:**
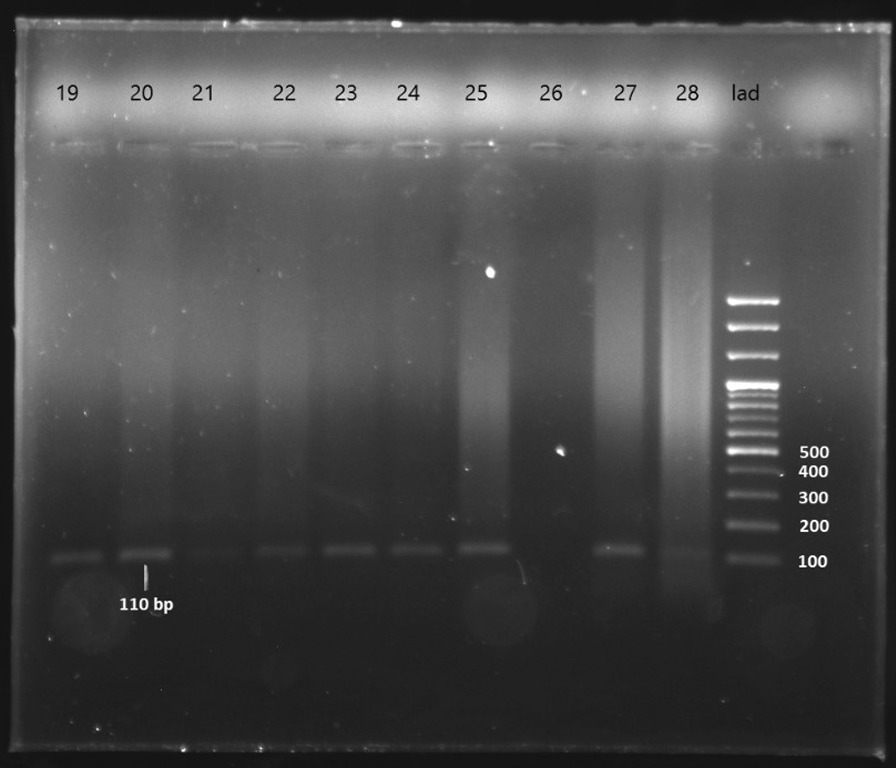
An agarose gel electrophoretogram of amplified GAPDH after the PCR as described in the materials and methods. Lanes 19 to 28 breast cancer samples, last lane: 100-bp DNA ladder

After extracting DNA from 59 FFPE breast cancer samples, and 11 benign breast lesions as control, we used PCR for the GAPDH gene to check the quality of DNA samples. The amplification of the GAPDH gene (fragment size of 110 bp) was positive in all extracted DNA, indicating an adequate quality of DNA. (Fig. 1).

### Detection of HPV and herpes viruses by PCR analysis

**Fig. 2:**
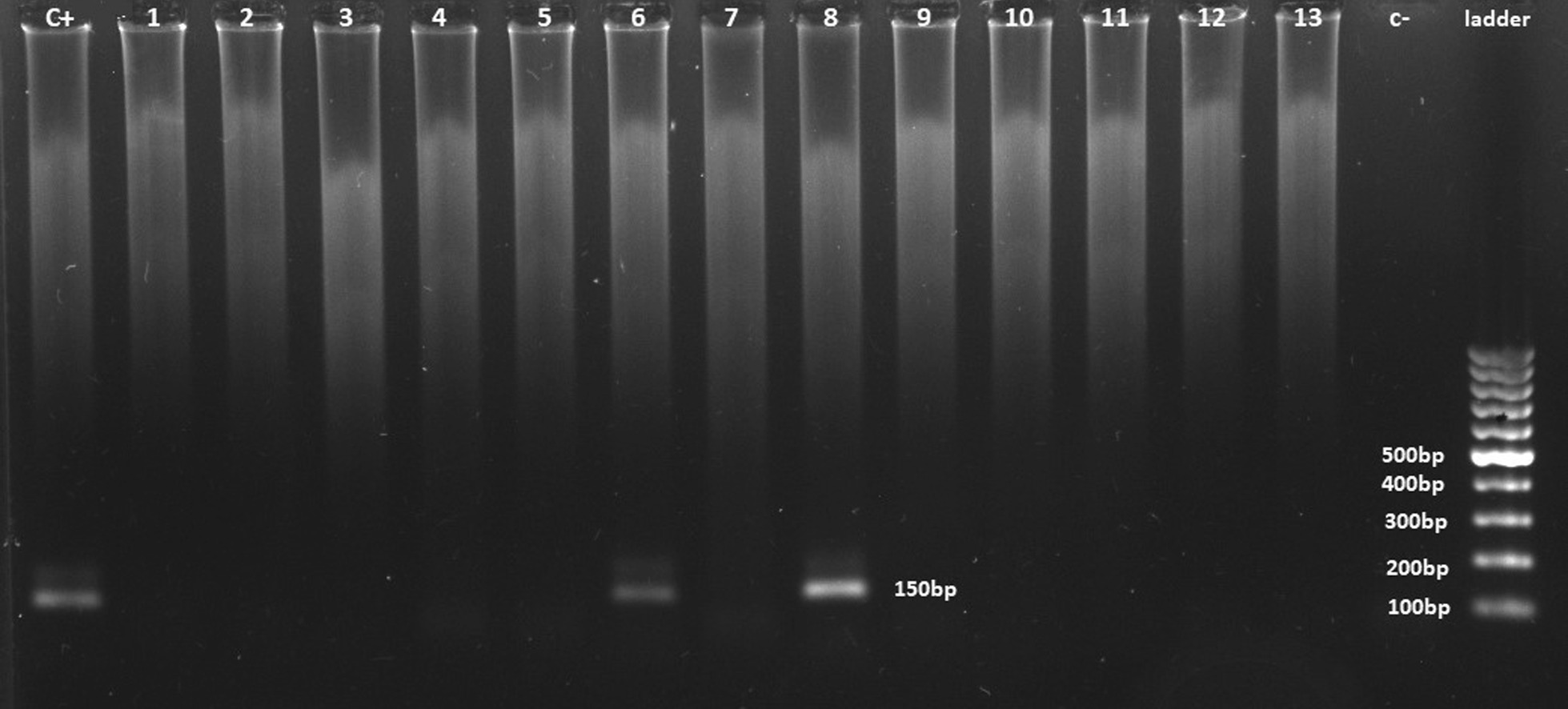
An agarose gel electrophoretogram of amplified DNA after the nested PCR as described in the materials and methods. C+ Lane: positive control (hpv18 HeLa cell line), Lanes 1 to 13 breast cancer samples, C− lane: negative control, last lane: 100-bp DNA ladder

7 of the 59 BC samples (11.8%) were positive for HPV DNA. The outer consensus primers MY09/MY11, and GP5+ /GP6+ inner primers were used in nested PCR. The length of products from the first and second steps of nested PCR was 450 bp and 150 bp respectively. None of the control samples were positive for HPV DNA (Fig. 2).

In our study, the age range of HPV positive patients was between 33 and 73 years (average age 51). All HPV positive patients had high tumor grades (II/ III). Histologic diagnosis of all positive cases was ductal carcinoma. Most of the patients had low levels of education (57.1%), 14.2% had no education and the remaining had a university degree (28.5%). (Table 3).

Analysis of real-time PCR supported the results of nested PCR and confirmed the presence of HPV18 in 5 breast tissue samples. HPV16 was not detected in any of the cases.

**Fig. 3:**
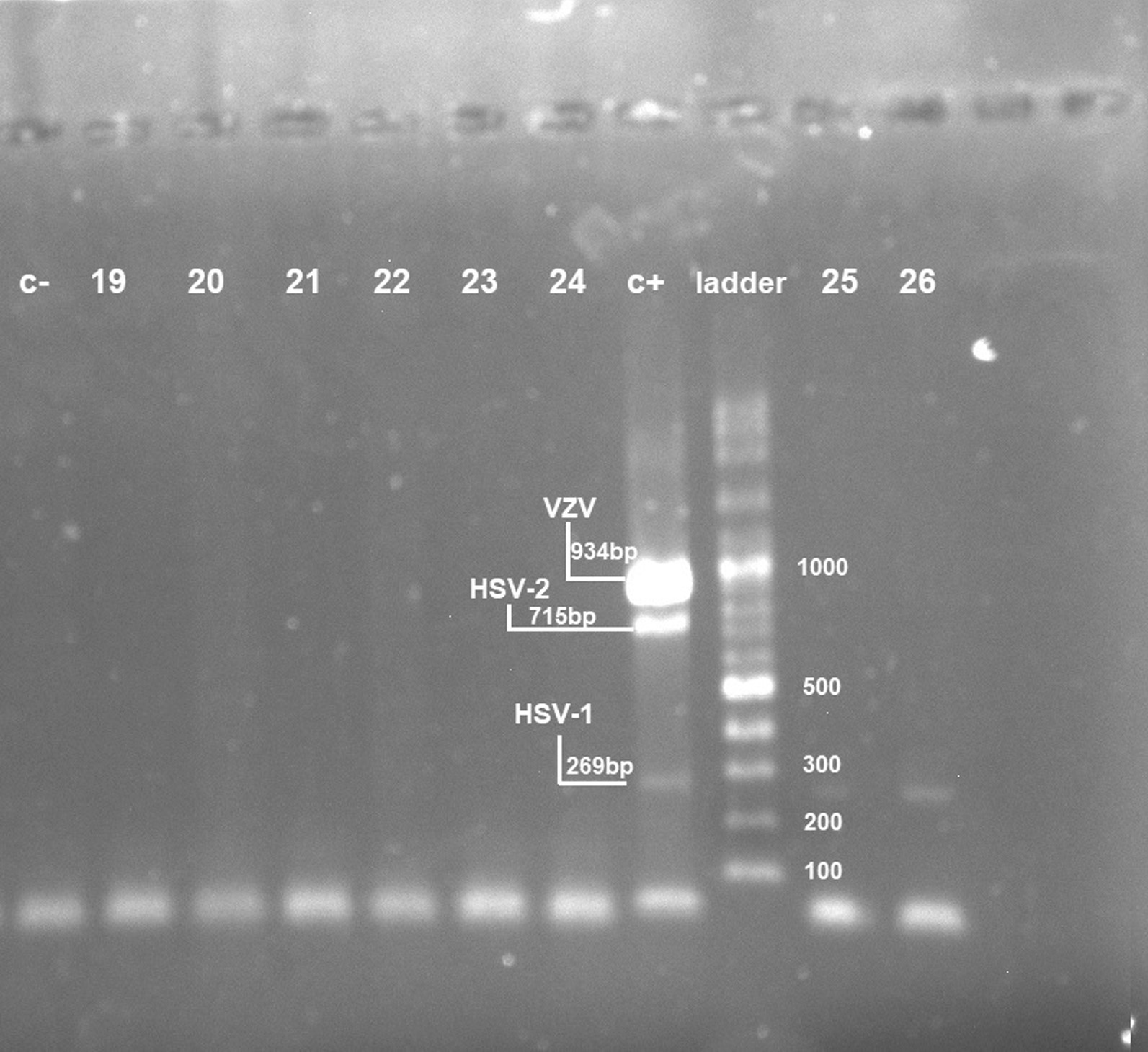
Gel electrophoresis of the multiplex PCR amplification products. Lanes 19 to 26 breast cancer samples. The amplicon length was 269 bp. C+ lane: positive control, C− lane: negative control, last lane: 100-bp DNA ladder

Herpes simplex 1 virus sequence was detected in 8.47% (5/59) of cases using mPCR (Fig. 3). None of the samples were positive for HSV-2, VZV, and CMV. The average age of HSV-1 positive patients was about 47 years. Most of the positive samples (80%) had high tumor grades (II/ III) (Table 3). Of the total of five cases, three had a low level of education, one had a university degree and one had no education. We verified the co-infection of HPV18 and HSV-1 in two of our cases (3.3%).

### Sequencing and construction of the phylogenetic tree

**Fig. 4:**
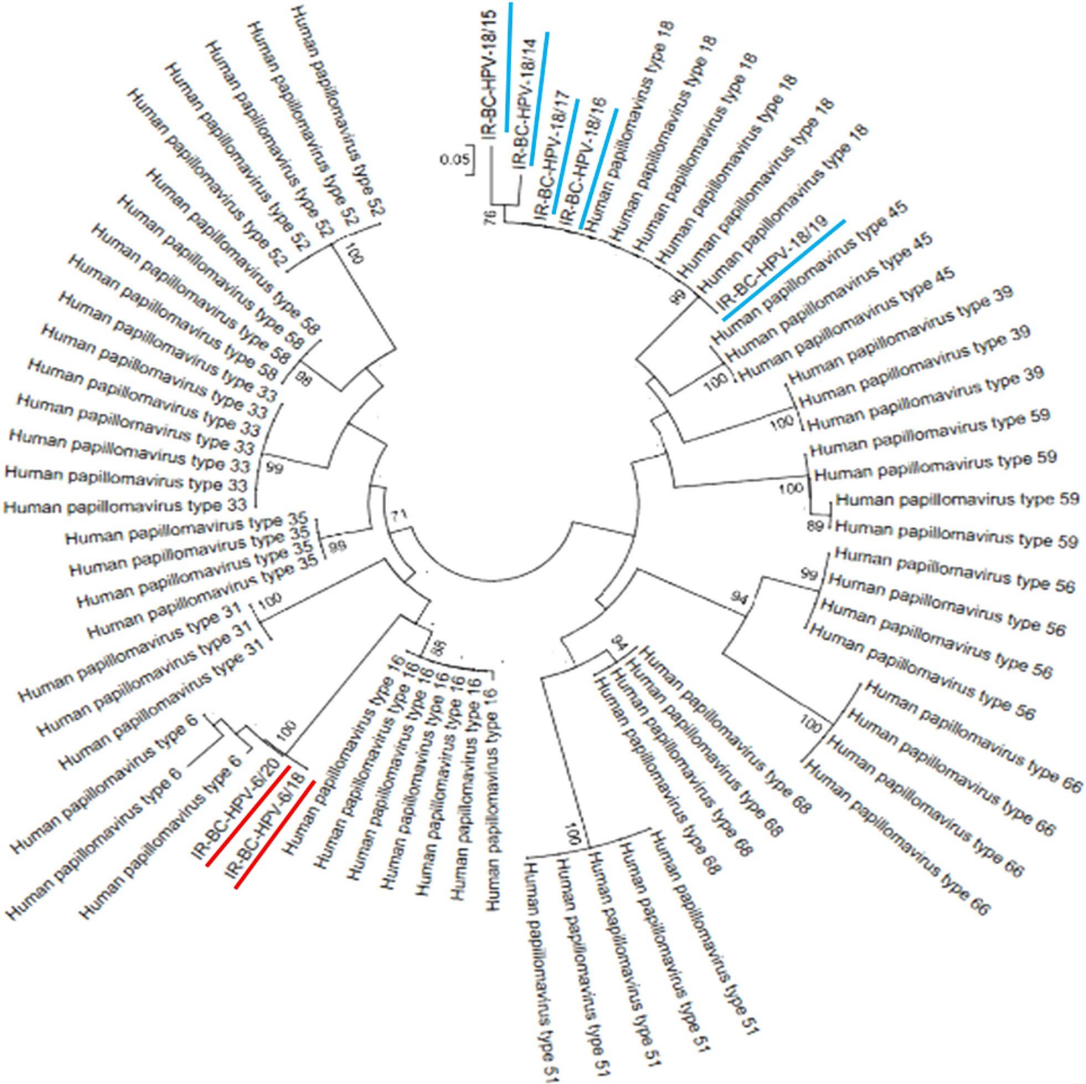
Phylogenetic trees of HPV16 and HPV18 Based on L1 Nucleotide were Constructed Using neighbor joining method and the Kimura 2-Parameter model by MEGA 6 package

All positive PCR products were sequenced. Five out of 7 HPV positive samples were HPV18 genotype (~ 72%), and the rest two (~ 28%) were HPV6 genotype Phylogenetic tree was shown in Fig. 4.

Based on our findings, the most prevalent type was HPV18, followed by HPV 6. Contrary to our expectations, we didn’t find any HPV16 genotype.

All of the HPV positive patients had invasive ductal carcinomas and high-grade tumors. The age range of 30 to 40 years had three positive samples; 2 of them were HPV18 and one of them was HPV6. The age range of 50 to 60 years had one HPV6 and one HPV18 positive samples. The age range of 60 to 70 years and 70 to 80 years, each with one HPV18 positive samples.

## Discussion

The worldwide increase in incidence and mortality of breast cancer is considerable [40]. This indicates the value of identifying responsible and preventable risk factors for breast cancer. Viral agents play a crucial role as carcinogens and they are associated with nearly 20% of cancers. The development of molecular techniques and subsequent investigations has proven the carcinogenic effect of high-risk HPV in various tumors, including breast carcinoma [41]. Based on published theories HR-HPVs trigger cancer by modifying cellular pathways by over-expressing E6 and E7 oncoproteins and suppressing the immune system [42, 43]. Numerous publications studied the association between HPV and cervical cancer [44–46]. But the correlation between HPV and breast cancer remains controversial. Lonardo A and his colleagues questioned the role of HPV16 in breast cancer for the first time in 1992 by identifying the DNA of this virus in 29.4% of BC samples [28]. Later, various studies were conducted worldwide. In 2011, according to a meta-analysis study, the prevalence of HPV in breast cancer in Europe was the lowest (12.91%), while was the highest in Oceania (42.11%) followed by Asia (32.42%) [47]. Based on their statistics and our findings, Iranian women with breast cancer are more vulnerable to HPV infection than European women but less vulnerable compared to Australian and North American women. One-fourth of Iranian women with BC are infected with HPV coincidentally. The prevalence of HPV infection in Iranian women with BC shows variation among cities/districts as follow: Shiraz (South city) 5.5%, Mazandaran (Northern district) 25.9%, Kermanshah (western district) 62.1%, Mashhad (Northeast city) 26.2%, and Tehran (Capital) 5.7%. [48–52]. In this study, we surveyed women with BC in Tehran and our result showed the 11.8% prevalence of HPV. Our results are similar to the findings of a study conducted in the United States in 2019, which used multiplex PCR with HPV E6 or E7 gene-specific primers for 64 serum samples of BC patients. So they reported 5 BC patients with positive HPV DNA (7.8%) [53]. Similarly, a study performed in 2011, detected 4 HPV DNA from 62 tumor tissue samples (6.5%) [54]. In contrast, different reports from the United States, the united kingdom, and Venezuela showed a higher percent of HPV prevalence in BCs; such as 86%, 42%, and 41.6%, respectively [29, 55, 56]. On the other hand, several studies performed in Denmark, Australia, India, etc. couldn’t find any association between the presence of HPV and breast cancer [32, 33, 57]. Some studies declared that HPV6 (low-risk HPV) was the most common type of virus among their specimens [30, 58] since we also found HPV6 in our samples so we recommend considering low-risk HPVs in breast cancer studies. Our result was similar to N Khodabandehlou et al. [59] and JS Lawson et al. [60], which reported HPV18 as the most prevalent type in positive samples. Noticeably, we did not find HPV type 16, which was demonstrated to be prevalent in previous studies [61, 62].

Differences in results may be due to variation in patient age range, the geographical distribution of viruses, techniques, and the quality of samples used in each study. According to a few published papers, the mean age of breast cancer in Iranian women is between 47.95 and 54.6 years [63–66]. In our study, the age range of patients was from 29 to 81 years (mean of 50 years) while according to a study in 2008 in Syrian women, the age range was 26 to 66 (with a median age of 52) with a higher prevalence of HPV (61.06%) [67]. Also, in our study, the age range of HPV-positive patients was from 33 to 73 years with a viral prevalence of 11.8%, but in a study in Venezuela, the age range was 51 to 60 years with a viral prevalence of 41.67% [55]. Therefore, the age range can be considered as an important issue in the rate of viral detection.

BC is often labeled as a “welfare disease” because the incidence rate was reported higher in women in Western societies with higher social class and higher education. Of course, this can be related to timely diagnosis and regular checkups, as well as better training in this group [68].

But in 2017, J Bahk and colleagues conducted the age-period-cohort (APC) analysis in Korea. They reported the BC mortality was higher in women with higher education than in women with no education or a primary education during 1983–1992, and the reverse was true in 1993–2012 [69].

In this study, a low level of education was found in 56%, and no education in 20% of patients. And in HPV-positive patients, 57.1% had low levels of education and 14.2% had no education. Regarding our results, most BC patients and HPV positive patients had low levels of education. Therefore, it can be noted that the education, training, and socioeconomic level of individuals can play a significant role in the prevention and timely treatment of breast cancer.

In this study, regarding histologic types, 95% of the total tumor specimens were ductal carcinoma and 5% were lobular carcinoma. And all positive HPV samples were ductal carcinoma. Similarly, in 2019, N Khodabandehlou et al. showed ductal carcinoma as the most frequent type in their HPV-positive patients [59].

Also, in the UK base study, NA Salman reported a higher prevalence of HPVs in patients with invasive ductal carcinoma [56]. From these studies, it can be concluded that mammary ducts provide an entry point for HPV infection [70].

Few studies surveyed the relation of tumor grades and HPV positive breast cancers. As the study of C Kroupis and et al. recorded high-grade tumors in 70.6% of HPV-positive breast cancer cases [61]. In our study, all positive samples had a high-grade tumor.

Ni Li and colleagues stated a remarkable point about the type of samples collected for studies. They showed a higher prevalence of HPV DNA in FFPE samples than fresh samples [47]. According to their findings the age of specimen also may affect the prevalence of virus detection. Our results indicated that samples collected earlier (from 2018 to 2019) showed higher HPV positivity compared to older collected samples (from 2008 to 2018).

Besides, due to the important role of herpesviruses as a co-factor in the oncogenesis of BC [39, 71, 72], we decided to detect this virus in our specimens. In 2013, BM Khashman et al. detected HSV-1 Ag with 31.8% prevalence using direct immunofluorescence in FFPE breast tissues [36]. We suggest utilizing a different technique, since it may affect results. In the literature, several studies are investigating the relationship between HPV and herpesviruses [73, 74]. So we used multiplex PCR with different primers for HSV-1, HSV-2, CMV, and VZV. We detected only HSV-1 with an 8.47% prevalence. Using an individual immunofluorescence method for each virus might be more specific and provide higher accuracy.

These data determine further evidence of a possible role for Human papillomavirus and Herpesvirus in breast carcinogenesis, however additional studies in a larger patient population and by techniques with higher specificities can ascertain our results. Also for early detection of cervical cancer, the American Cancer Society guidelines recommend Pap test and HPV DNA testing. Currently, the American Cancer Society recommends mammography for regular breast cancer screening, while further studies on the association between breast cancer and HPV in the future may add HPV screening to this guideline [75].

## Conclusion

Overall, in the present study, we identified HPV18 and HPV6 genotypes in 11.8% and HSV-1 in 8.47% of FFPE samples from women with breast cancer.

HPV18 was the most common genotype. And coinfection of HPV and HSV-1 was declared in 3.3% of them. Our conclusion suggests a carcinogenesis role for HPV and HSV-1 in breast cancer patients, but it is required to study more to find these viruses' role in Breast cancer.

## Data Availability

The datasets used and/or analyzed during the current study are available from the corresponding author on reasonable request.

## Abbreviations

HPV: Human papillomavirus
BC: Breast cancer
HSV: Herpes simplex virus
CMV: Cytomegalovirus
VZV: Varicella zoster virus
HR: High risk
LR: Low risk
FFPE: Formalin-Fixed Paraffin-Embedded
PCR: Polymerase chain reaction
mPCR: Multiplex polymerase chain reaction
GAPDH: Glyceraldehyde-3-phosphate dehydrogenase
bp: Base pair
min: Minute
